# Considering Usual Medical Care in Clinical Trial Design

**DOI:** 10.1371/journal.pmed.1000111

**Published:** 2009-09-29

**Authors:** Liza Dawson, Deborah A. Zarin, Ezekiel J. Emanuel, Lawrence M. Friedman, Bimal Chaudhari, Steven N. Goodman

**Affiliations:** 1 Division of AIDS, National Institute of Allergy and Infectious Diseases, National Institutes of Health, Bethesda, Maryland, United States of America; 2 ClinicalTrials.gov, National Library of Medicine, National Institutes of Health, Bethesda, Maryland, United States of America; 3 Department of Clinical Bioethics, Warren G. Magnusen Clinical Center, National Institutes of Health, Bethesda, Maryland, United States of America; 4 Independent Consultant, Rockville, Maryland, United States of America; 5 Department of Pediatrics, Washington University School of Medicine, St. Louis, Missouri, United States of America; 6 Department of Oncology, Johns Hopkins University School of Medicine, Baltimore, Maryland, United States of America

## Abstract

Liza Dawson and colleagues discuss the scientific and ethical issues associated with choosing clinical trial designs when there is no consensus on what constitutes usual care.

**Summary Points**

- Challenges often arise when researchers propose clinical trials incorporating usual care comparison groups.
- Disagreements may arise about current levels of evidence supporting usual care or failures to use best known methods in clinical practice; about the need for customized care, or about the difficulty in choosing best treatments when available interventions have trade-offs.
- Clinical trial designs incorporating usual care arms must be based on scientific validity, consideration of risks and benefits to patients, relevance to the clinical care community, and feasibility.

## Introduction

In 2002, a clinical trial designed to evaluate optimal ventilation practice [1],[2] for patients with acute respiratory distress syndrome (ARDS) sparked a major controversy. Critics charged that management of ARDS in the different arms of the study did not adequately reflect usual medical care, and alleged that it was essential for scientific and ethical reasons to have a usual care comparison arm in the study. The controversy over trial design enmeshed the National Institutes of Health (NIH), the Office for Human Research Protections (OHRP) and the critical care research community. The trial was put on hold and reviewed by two independent expert panels. Experts pointed to the need for further analysis of the scientific and ethical issues involved in choosing trial designs when there is no consensus on standard of care.

In November 2005, NIH and a number of other federal agencies sponsored a meeting (see Text S1) to discuss clinical trial design challenges involving selection of usual care comparison groups (Text S1). The meeting was informed by a background paper (Text S1) outlining types of challenges involved in selecting usual care arms, prepared by a working group with expertise in clinical trial design, ethics, evidence-based medicine, statistics, and science policy. We present here the background framework and case studies used in this paper (Text S1). We enumerate five factors that make consensus on these issues particularly difficult, and recommend specific criteria for assessing proposed study designs.

### Terminology

Terms such as “standard of care,” “control arm,” “usual care,” and “community care” have all been used to describe arms reflecting conventional therapy. We use the term “usual care” to describe the care commonly given by practitioners in a community to avoid any legal or normative implications of the term “standard of care.”

## Determining When a Usual Care Arm Will be Needed

There may be scientific, ethical, and/or practical reasons for having an arm in a clinical trial that employs usual care. If researchers hypothesize that a new intervention is better than or at least equivalent to current clinical practice, then one trial arm needs to reflect usual care. Ethically, the clinical care community must be in a state of equipoise prior to randomizing patients to different interventions [3], although there is no universal view on how to evaluate or resolve disagreements on the existence of equipoise in a particular scenario. If clinicians or investigators believe that usual care is effective, a usual care comparison may increase trial acceptability. A usual care arm might improve relevance, external validity, or the practicality of the study.

## Challenges in Formulating Comparison Groups Representing Current Medical Care

Five types of difficulties can arise in defining a comparison group, and several of these conditions often coexist: (1) disputes about evidence; (2) low level of utilization of best methods; (3) trade-offs relating to physician and patient preferences for different treatments; (4) an insufficient preexisting evidence base to guide treatment selection; and (5) individually customized medical care for conditions with no standard practice guidelines.

Underlying these issues are two fundamental tensions. First, there is tension between the need for control over experimental conditions and the need for trials to be relevant to clinical care in the community. This tension has been described as a distinction between pragmatic and explanatory trials [4], between explanatory and management trials [5], or between mechanistic and practical trials [6]. It may be difficult to interpret data from trials that incorporate the most relevant, and often highly variable, clinical practices; for example, when fundamentally different treatments are combined in a single arm, bias or confounding may exist within an arm. Conversely, a more tightly controlled experiment may not yield information that is widely applicable or considered relevant.

Second, lack of consensus on the current evidence base confounds attempts to design new trials. Trials should build upon previous evidence and address gaps in knowledge, but achieving this goal depends upon some agreement among stakeholders about interpretation of the state of current evidence and priorities for research.

## Disputes about Interpretations of Evidence

Experts may disagree about interpretation of the available evidence and about whether current treatments have been validated by research (Box 1). This lack of consensus on which treatments should be considered “standard” can lead to divergent views on the selection of a comparison group, and more fundamentally, dispute about what research question is most relevant [7],[8].

**Box 1. Case Example: Taxanes and Ovarian Cancer Treatment**

Before taxanes were available, first-line treatment for advanced ovarian cancer consisted of carboplatin, either alone or in combination with other drugs. In the early 1990s, four large trials were undertaken to determine if the addition of taxanes could improve survival in patients with advanced disease [37]–[40]. Two trials showed a survival benefit for patients on paclitaxel-containing regimens, while two trials revealed no significant differences. One commentator [41] outlined different explanations for the divergent trial results, such as differences in the extent of treatment crossover among trials, differences in patients, and differences in control arms. Experts in the US considered the positive trials to be definitive, while those in the UK believed the trials showing equivalence carried more weight.

Consequently, in an international collaboration involving the US, UK, and Canada, national differences in practice guidelines—based on divergent views of the evidence—led to disagreements about the appropriate reference arm in a trial adding newer drugs to existing regimens. In the trial, Gynecologic Oncology Group 182-International Collaborative Ovarian Neoplasm (ICON) 5 [42], the UK investigators advocated for flexibility in the comparison group, due to their view that taxane-containing regimens were equivalent to older regimens, but the US investigators believed that paclitaxel must be included in first-line treatment. In the end, the reference arm in the trial consisted solely of the paclitaxel-containing regimen, and flexibility was not allowed [43].

Designs that directly address the source of the evidentiary controversy are valuable, but it might be impossible to design a study that is acceptable to all. Experts may disagree about whether there is sufficient uncertainty to conduct a trial, or about the risk–benefit profile of any particular design. Some might believe that evidence already exists that a particular intervention is inferior or poses serious risks; others who believe that evidence is not clear might advocate for a trial to compare competing interventions.

In these situations, the most important first step is to correctly identify the source of disagreement about evidence, which can then be a focus of discussion.

## Lack of Adherence to Evidence-Based Recommendations or Practice Guidelines and Other Variations in Medical Practice

Proven interventions may not be widely used [9] because of low physician confidence or knowledge, difficulty in implementation, cost, side effects, or patient heterogeneity.

The choice of research question and study design may depend on an analysis of the factors driving the low utilization. Disagreements can arise about whether a validated treatment that is not used in the community should be considered standard and provided to a control group in a trial (Box 2). If usual medical practice is used as a comparator arm, it may expose subjects to less than optimal medical care; some might defend such a design on the basis of common practice in the community and societal benefit from knowledge to be gained. The acceptability of this approach depends in part on whether there is a possibility of serious or irreversible harm to patients receiving usual care.

**Box 2. Case Example: The Enhanced Suppression of the Platelet IIb/IIIa Receptor with Integrilin Trial (ESPRIT) Trial**

ESPRIT was designed to determine the efficacy of a platelet glycoprotein (GP) receptor antagonist, eptifibatide (Integrilin) in reducing the incidence of various coronary events in percutaneous coronary intervention (PCI). During study planning there was a vigorous debate about whether the trial should be have a placebo or active control, namely abciximab [9],[44]. In spite of evidence from previous studies indicating positive effects of abciximab in PCI, this agent was not used in 65%–75% of PCI procedures. Reasons for low usage were clinician concerns about cost, safety, and efficacy; some physicians had doubts about the applicability of previous trial data to current uses.

The FDA challenged the placebo-controlled study design [45]. A survey of investigators at 49 ESPRIT sites revealed that only 30% used platelet GP IIb/IIA inhibitors in management of PCI patients, and a substantial proportion of these used the drugs in bail-out treatment. With these data the FDA and investigators felt it was ethical to utilize a placebo control arm because it would not be withholding from research participants a treatment they would otherwise receive, although both the FDA and the investigators thought “usual care” was potentially inferior to best practices.

Where the prevailing practice is no treatment, investigators might consider a placebo control, but may be constrained by ethical demands for an active comparison group. There are existing guidelines for the use of placebos [10],[11] that define specific criteria for their use.

If researchers test a new intervention that could match the effectiveness of the gold standard but is cheaper, easier, or more accessible, it would be reasonable to use the best known method as a comparator in a noninferiority design. However, if the new method is likely to be inferior to the best known treatment but better than the usual care patients actually receive, a quandary remains: which existing method should be used as a comparator?

Generally, noninferiority trials require greater numbers of subjects than do superiority trials, If a new intervention is compared to best methods, the feasibility of conducting the noninferiority trial might be a limiting factor in getting the research off the ground. A superiority trial using an inferior reference arm might be more feasible but objectionable because of the less than optimal comparison group. There is no consensus on how these situations should be handled.

A trial might be designed as a strategy trial to test an intervention delivered according to a specific algorithm head-to-head against the same intervention as used in the community. The acceptability of this design might depend on whether the best-practices algorithm is widely considered more effective, or whether this is still an open question.

An example of such a trial is the Hypertension Detection and Follow-up Program [12], which compared the effect of Stepped Care versus community medical therapy, with the primary endpoint being five-year all-cause mortality. This landmark study found that an intensive management algorithm for hypertension treatment improved outcomes, compared to community care. It is interesting to note that certain secondary outcomes could not be assessed without bias, because of the nature of the comparison arms. For example, events diagnosed by direct observation, such as nonfatal myocardial infraction, were not bias-free endpoints due to the closer monitoring of the Stepped Care arm compared to community care. Therefore, all-cause mortality was the sole primary endpoint. It is also notable that research center staff took direct steps to ensure that patients in the community care arm with higher levels of hypertension or major organ system abnormalities were seen by a community provider.

## There Is No Single “Best” Treatment: Different Treatments Have Trade-offs in Terms of Different Outcomes or Side Effects

Two or more treatments for a single condition may be characterized by different profiles of performance across different measures or side effects. Regimens can be chosen on the basis not only of effectiveness but also side effects or quality of life [13],[14]. Treatment choices may be made on the basis of disease or patient characteristics, on physician or patient preferences, or all of these factors (Box 3).

**Box 3. Case Example: The Multi-modal Treatment Study of ADHD (MTA)**

The MTA [46]–[48] exhibited some features of the “gold standard” versus community care approach. The main research question was about the relative efficacy of drug treatment, behavioral treatment, or a combination of the two. Therefore, the medication management, behavioral, and combination interventions were carefully structured according to best practices to give what investigators hoped would be the optimal results for each modality. Medication management involved careful adjustment of dosage and choice of medication, medication three times daily, and monthly follow-up visits and support. Intensive behavioral treatment consisted of eight individual meetings interspersed with 27 group meetings to teach parents behavioral management techniques, an intensive 8 week summer program for children, and classroom behavioral aides during the fall of the school year. The third arm combined the medication and behavioral interventions. A fourth arm consisted simply of referral to care in the community, with follow-up and data collection in parallel with the other three assigned treatment arms. Hence, the study included features of both explanatory and pragmatic trials.

While the main research question in MTA was not about the adequacy of usual care, the inclusion of the community care arm allowed some important data to be collected about the effectiveness of usual care practices compared to the intensive, carefully monitored interventions delivered in the other three trial arms. Detailed data collection on procedures in the usual care arm informed further work on translating the clinical trial results back into community practice [49].

When available treatments present trade-offs, patient preferences are often particularly relevant [15]–[18]. A classically randomized trial may be hindered by a high refusal rate at recruitment or by significant unplanned crossover between or among arms after randomization. Some investigators have explored partially randomized designs that include randomized groups and an observational arm in which patients choose treatments [19] (Box 4). Another option is testing a single treatment versus a usual care arm allowing patient and provider choice. This may increase the relevance of the trial and enhance participation. However, as in other not completely randomized studies, inferences that can be made from a heterogeneous patient preference arm are limited by possible biases and confounding.

**Box 4. Case Example: The Spine Patient Outcomes Research Trial (SPORT)**

SPORT randomized patients to surgical versus nonsurgical treatments for back pain [19]. Patients in the nonsurgical treatment arm were free to choose among a long list of treatment alternatives. One of the strengths of this trial design is that the wide range of practices used in the community were systematically documented in the trial, rather than used covertly in a trial where only a subset of available treatments are permitted and where patients may seek additional care outside the trial itself.

## Lack of, or Insufficient, Evidence Base for Existing Treatments

Often, treatments used in clinical practice have been insufficiently evaluated in rigorous clinical trials. This problem may occur with non-drug interventions or with drugs that have not been tested against relevant comparators. Clinical trial data may be scanty, of poor quality, or based on irrelevant patient populations; many treatments have not been systematically evaluated in randomized clinical trials (RCTs) [20]–[22]. With this lack of evidence it may not be clear which treatment is preferable, or even if a given treatment is better or worse than nothing.

Trials addressing these kinds of evidence gaps could be designed with multiple arms comparing existing interventions or comparing a single intervention to a heterogeneous group of treatments in the “usual care” arm.

The principal problem with this flexible usual care group design is the limitations on inferences that may be drawn unless the single intervention is clearly superior. In noninferiority trials, inferences could be problematic if there is a lack of solid evidence supporting effectiveness of a usual care arm [23]. Also, heterogeneity in the usual care group may make it difficult to interpret and apply the results.

## Physician Attitudes Regarding Customized Patient Care

Selection of customized treatment based on physician assessment of individual patient characteristics [24] can lead to scientific and practical challenges in measuring effectiveness in clinical trials [25]. When many patient characteristics are relevant, it would require impossibly large trials to encompass all the stratified patient subgroups needed to individually test all the factors used in decision-making. In such situations, physicians may object to protocolized usual care treatment groups in clinical trials, based on a belief that physician discretion in treatment choices provides superior outcomes [26]–[33]. In addition, data, especially from explanatory trials, come from carefully selected populations that differ in major ways from patients treated in the community.

Physician decision-making can be tested in a flexible usual care arm, although if physicians vary in their criteria for assigning individual treatments, it will be impossible to make inferences about which set of criteria is best. A preferable alternative is to test disease management algorithms versus usual practices [34],[35].

## Discussion

The choice of comparison arms in clinical trials can be challenging when there is no clear-cut uniform standard of care. A variety of non-mutually exclusive factors can feed the lack of consensus: differing interpretations of existing evidence, inadequate evidence, different balancing of trade-offs, a failure or inability to implement evidence-based therapies, or a belief in customized care.

It is critical to think systematically about the background conditions in the practicing medical community and goals of the trial when grappling with the complexities of heterogeneous medical practices. Multiple research questions could be important, each requiring a different trial design. At a minimum, the background conditions of medical practices and beliefs should be thoroughly explored, sometimes with qualitative as well as quantitative research.

Potential trial designs should be examined based on the following criteria:

- Scientific validity and strength of inferences possible from a given design;
- Risks and benefits to participants in chosen design versus alternative designs;
- Relevance of the trial to current practice, including relevance to provider and patient beliefs and values;
- Feasibility of the trial.

If a usual care arm is proposed, the scientific rationale for including such an arm should be carefully evaluated. It is critical to consider whether the usual care arm will contribute to meaningful inferences about the relative merits of different interventions in the trial, and whether the protocol should restrict or intervene in usual care. Design choices regarding protocolized versus unrestricted usual care often involve navigating a tension between the need for rigor and clarity of evidence versus practicality and relevance to clinical practice.

If less than best accepted medical care is provided in a trial arm it must be carefully evaluated and justified. When there are disputes about the adequacy of, or evidence base for, any of the interventions proposed for the trial, there may be no consensus on whether trial participants are adequately protected—these disagreements about evidence should be frankly acknowledged.

The relevance of the trial to current practice should be described. Finally, practical limitations should be acknowledged, including infrastructure, costs, willingness to participate, time constraints, or other factors.

Not all “usual care” trials have similar purposes. The SPORT trial (Box 4) defines one end of the spectrum: a usual-care arm that consists of a heterogeneous mix of practices that are not mechanistically related. The result from such a trial might be questioned as uninterpretable because the comparator to the surgery intervention is not defined. However, this trial is a useful exploration, providing evidence on a potpourri of treatments that could help refine the comparisons made in a future trial. Viewed from this perspective, the trial is akin to a high-quality observational study, with randomization reducing, but not eliminating, the confounding introduced by patient or physician choice. The trial then is helpful as part of a series of studies in which no single study is definitive. In fact, recently published results [36] reveal that due to extensive crossover between treatment arms, it is impossible to draw clear conclusions about relative effectiveness of surgery versus nonsurgical treatments from the trial results.

On the other end of the spectrum is the ovarian cancer trial, in which a dispute about the appropriate comparator was resolved with a choice of one treatment that was not yet universally used, but was viewed by some as best proven therapy. Such trials pose no problems of interpretability. Trials that occupy an intermediate category are those that use multiple arms that implement different therapeutic approaches used in practice, but that share a common mechanism, such as different degree of the same therapy. In such trials, the pattern of results among the arms becomes relevant, as either a flat or monotonic dose–response is expected. The arms therefore “borrow strength” from each other in ways that mechanistically heterogeneous treatment choices or combinations cannot.

Choices of control or comparator conditions can become surrogates for debates about the adequacy of current medical practice, about current scientific evidence, or about assessment of trade-offs among treatment options. These debates can affect judgments about whether sufficient uncertainty exists to conduct the trial at all; whether risks to subjects are minimized; and whether the trial data will be interpretable. Disputes about background conditions complicate these already difficult discussions, and new empirical data on practice patterns can help clarify such debates. What is critical in all of these situations is that the reasons for disagreement about usual care be recognized and addressed separately from the question of the trial design.

The goal should be that each trial will contribute to the accumulation of knowledge via a sequence of investigations, which together lead to a causally coherent understanding of treatment effects. Ultimately, we want to answer why a treatment is effective, by how much versus a defined comparator, at what risk, and in which patients. So an investigator must be able to look beyond the trial in question and explain how its results will inform future research that lead to such an understanding. Studies implementing “usual care” arms can complicate this task, but if done right can ultimately lead to results of great scientific relevance and practical value.

## Supporting Information

Text S1 Considering usual medical care in clinical trial design: Scientific and Ethical Issues Meeting, November 2005, Bethesda, Maryland. In November 2005, NIH and a number of other federal agencies sponsored a meeting to discuss clinical trial design challenges involving selection of usual care comparison groups. The planning committee for the meeting consisted of the following individuals: Duane Alexander, NIH/NICHD; Jonathan Berman, NIH/NCCAM; Carolyn Clancy, AHRQ; Ezekiel Emanuel, NIH/Clinical Center; Ellen Feigal, NIH/NCI; Lawrence Friedman, NIH/NHLBI; John Gallin, NIH/Clinical Center; Saul Malozowski, NIH/NIDDK; Peter Mannon, NIH/NIAID; Joan McGowan, NIH/NIAMS; Amy Patterson, NIH/OD; Marcel Salive, CMS; Bernard Schwetz, OHRP; Belinda Seto, NIH/OER; David Shore, NIH/NIMH; Lana Skirboll, NIH/OD; Robert J. Temple, FDA; Deborah Zarin, AHRQ. The meeting was informed by a background paper outlining types of challenges involved in selecting usual care arms, prepared by a working group with expertise in clinical trial design, ethics, evidence-based medicine, statistics, and science policy. The drafting group for the background paper consisted of Liza Dawson, Ezekiel Emanuel, Lawrence Friedman, Steven Goodman, and Deborah Zarin. At the meeting, case study presentations were made by Taylor Thompson, Mass. General Hospital, Acute Respiratory Distress Syndrome Network (ARDSnet); Ann Marie Swart, UK Medical Research Council, International Collaborative Ovarian Neoplasm (ICON) Trials; James Swanson, UC Irvine, Multimodal Treatment Study of ADHD (MTA); James Weinstein, Dartmouth Medical School, Spine Patient Outcomes Research Trial (SPORT). A full presentation of each case study and panel discussion is included in the meeting proceedings document at http://crpac.od.nih.gov/Draft_UsualCareProc_06062006_cvr.pdf.
(0.03 MB DOC)

## Abbreviations

ARDS: acute respiratory distress syndrome
NIH: National Institutes of Health
OHRP: Office for Human Research Protections
PCI: percutaneous coronary intervention
PG: platelet glycoprotein
RCT: randomized controlled trial
