# Supplementary material for: Considering Usual Medical Care in Clinical Trial Design
Source: PLoS Med. 2009 Sep 29;6(9):e1000111. doi: 10.1371/journal.pmed.1000111 (PMC2746285; doi:10.1371/journal.pmed.1000111)
Supplement: Text S1 — Considering usual medical care in clinical trial design: Scientific and Ethical Issues Meeting, November 2005, Bethesda, Maryland. In November 2005, NIH and a number of other federal agencies sponsored a meeting to discuss clinical trial design challenges involving selection of usual care comparison groups. The planning committee for the meeting consisted of the following individuals: Duane Alexander, NIH/NICHD; Jonathan Berman, NIH/NCCAM; Carolyn Clancy, AHRQ; Ezekiel Emanuel, NIH/Clinical Center; Ellen Feigal, NIH/NCI; Lawrence Friedman, NIH/NHLBI; John Gallin, NIH/Clinical Center; Saul Malozowski, NIH/NIDDK; Peter Mannon, NIH/NIAID; Joan McGowan, NIH/NIAMS; Amy Patterson, NIH/OD; Marcel Salive, CMS; Bernard Schwetz, OHRP; Belinda Seto, NIH/OER; David Shore, NIH/NIMH; Lana Skirboll, NIH/OD; Robert J. Temple, FDA; Deborah Zarin, AHRQ. The meeting was informed by a background paper outlining types of challenges involved in selecting usual care arms, prepared by a working group with expertise in clinical trial design, ethics, evidence-based medicine, statistics, and science policy. The drafting group for the background paper consisted of Liza Dawson, Ezekiel Emanuel, Lawrence Friedman, Steven Goodman, and Deborah Zarin. At the meeting, case study presentations were made by Taylor Thompson, Mass. General Hospital, Acute Respiratory Distress Syndrome Network (ARDSnet); Ann Marie Swart, UK Medical Research Council, International Collaborative Ovarian Neoplasm (ICON) Trials; James Swanson, UC Irvine, Multimodal Treatment Study of ADHD (MTA); James Weinstein, Dartmouth Medical School, Spine Patient Outcomes Research Trial (SPORT). A full presentation of each case study and panel discussion is included in the meeting proceedings document at http://crpac.od.nih.gov/Draft_UsualCareProc_06062006_cvr.pdf. (0.03 MB DOC) [file pmed.1000111.s001.doc]

Text S1

Dawson et al.

Considering Usual Medical Care in Clinical Trials: Scientific and Ethical Issues

The planning committee for the meeting consisted of the following individuals: Duane Alexander, NIH/NICHD; Jonathan Berman, NIH/NCCAM; Carolyn Clancy, AHRQ; Ezekiel Emanuel, NIH/Clinical Center; Ellen Feigal, NIH/NCI; Lawrence Friedman, NIH/NHLBI; John Gallin, NIH/Clinical Center; Saul Malozowski, NIH/NIDDK; Peter Mannon, NIH/NIAID; Joan McGowan, NIH/NIAMS; Amy Patterson, NIH/OD; Marcel Salive, CMS; Bernard Schwetz, OHRP; Belinda Seto, NIH/OER; David Shore, NIH/NIMH; Lana Skirboll, NIH/OD; Robert J. Temple, FDA; Deborah Zarin, AHRQ.

The meeting proceedings document is available at <http://crpac.od.nih.gov/Draft_UsualCareProc_06062006_cvr.pdf>.

The drafting group for the background paper consisted of Liza Dawson, Ezekiel Emanuel, Lawrence Friedman, Steven Goodman, and Deborah Zarin.

Case study presentations were made by Taylor Thompson, Mass. General Hospital, Acute Respiratory Distress Syndrome Network (ARDSnet); Ann Marie Swart, UK Medical Research Council, International Collaborative Ovarian Neoplasm (ICON) Trials; James Swanson, UC Irvine, Multimodal Treatment Study of ADHD (MTA); James Weinstein, Dartmouth Medical School, Spine Patient Outcomes Research Trial (SPORT). A full presentation of each case study and panel discussion is included in the meeting proceedings document at <http://crpac.od.nih.gov/Draft_UsualCareProc_06062006_cvr.pdf>.
